# NADPH oxidase 4 signaling in a ventilator-induced lung injury mouse model

**DOI:** 10.1186/s12931-022-01992-0

**Published:** 2022-03-27

**Authors:** Sang Hoon Lee, Mi Hwa Shin, Ah Young Leem, Su Hwan Lee, Kyung Soo Chung, Young Sam Kim, Moo Suk Park

**Affiliations:** Division of Pulmonary and Critical Care Medicine, Department of Internal Medicine, Institute of Chest Diseases, Severance Hospital, Yonsei University College of Medicine, 50-1, Yonsei-ro, Seodaemun-gu, Seoul, 120-752 South Korea

**Keywords:** NADPH oxidase 4, Ventilator, Lung injury, EphA2, IL-6

## Abstract

**Background:**

For patients with acute respiratory distress syndrome, a ventilator is essential to supply oxygen to tissues, but it may also cause lung damage. In this study, we investigated the role of NOX4 using NOX4 knockout (KO) mice and NOX4 inhibitors in a ventilator-induced lung injury (VILI) model.

**Methods:**

Wild-type (WT) male C57BL/6J mice and NOX4 knockout (KO) male mice were divided into five groups: (1) control group; (2) high tidal ventilation (HTV) group: WT mice + HTV ± DMSO; (3) NOX4 KO group; (4) NOX4 KO with HTV group; (5) NOX4 inhibitor group: WT mice + HTV + NOX4 inhibitor. In the VILI model, the supine position was maintained at 24 mL/kg volume, 0 cm H_2_O PEEP, 100/min respiratory rate, and 0.21 inspired oxygen fraction. In the NOX4 inhibitor group, 50 μL anti-GKT 137831 inhibitor was injected intraperitoneally, 2 h after ventilator use. After 5 h of HTV, mice in the ventilator group were euthanized, and their lung tissues were obtained for further analysis. In addition, the relationship between EphA2 (which is related to lung injury) and NOX4 was investigated using EphA2 KO mice, and NOX4 and EphA2 levels in the bronchoalveolar lavage fluid (BALF) of 38 patients with pneumonia were examined.

**Results:**

Cell counts from BALFs were significantly lower in the NOX4 KO with HTV group (p < 0.01) and EphA2 KO with HTV group (p < 0.001) compared to that in the HTV group. In the NOX4 inhibitor group, cell counts and protein concentrations from BALF were significantly lower than those in the HTV group (both, p < 0.001). In the NOX4 KO group and the NOX4 inhibitor group, EphA2 levels were significantly lower than those in the HTV group (p < 0.001). In patients with respiratory disease, NOX4 and EphA2 levels were significantly higher in patients with pneumonia and patients who received ventilator treatment in the intensive care unit.

**Conclusion:**

In the VILI model with high tidal volume, NOX4 KO, EphA2 KO or monoclonal antibodies attenuated the VILI. NOX4 and EphA2 levels were significantly higher in patients with pneumonia and especially in mechanical ventilated in the ICU. Inhibition of Nox4 is a potential therapeutic target for the prevention and reduction of VILI.

**Supplementary Information:**

The online version contains supplementary material available at 10.1186/s12931-022-01992-0.

## Introduction

Acute respiratory distress syndrome (ARDS) is defined as a syndrome involving severe hypoxia (PaO_2_/FiO_2_ ≤ 200 mmHg) with bilateral pulmonary infiltration in radiologic findings and non-cardiogenic pulmonary edema. Acute lung injury (ALI) is defined by less severe hypoxemia (PaO_2_/FiO_2_ ≤ 300 mmHg) [1–4]. In 2003, Goss et al*. *[5] reported a prevalence of 17.6–64.0 ALIs per 100,000 people based on American hospital association data, and, in 2005, Rubenfeld et al*. *[6] reported 78.9 ALIs per 100,000 people by applying the American-European Consensus Conference (AECC) criteria for the determination of respiratory distress.

The use of low tidal volume ventilation in ARDS as well as the application of established protocols for sepsis or pneumonia treatment, computerized data, and sufficient medical workforce may all have led to the reduced incidence of ARDS/ALI. However, there has been no change in the fundamental treatment of ARDS/ALI beyond an improvement in intensive care unit (ICU) quality management [7–9], and, as populations age, ARDS/ALI will become a greater social and economic problem [6]. Therefore, it is important to determine the pathophysiology of ARDS/ALI through animal experiments and to identify the molecular mechanisms that underlie ARDS/ALI, in order to develop new therapeutic agents for these conditions.

Correcting hypoxemia through artificial respiration is an important therapy used to treat patients with ARDS/ALI, but the ventilator itself may cause lung injury and exacerbate existing ARDS/ALI [10]. Therefore, it is important to analyze the mechanism of pulmonary damage by ventilators to modify clinical management of hypoxemic patients.

In the lungs, NADPH oxidase 4 (nicotinamide adenine dinucleotide phosphate-oxidase 4, NOX4) has been studied mainly because of its association with lung cancer. Zhang et al*. *[11] showed that there was good agreement between the level of NOX4, cancer stage, and survival time in non-small-cell lung cancer (NSCLC). They also reported that overexpression of NOX4 in A549 cells promoted their proliferation and invasion, increased tumor size, shortened survival time, and stimulated lung metastasis when compared with that in the control group. Conversely, depletion of NOX4 has been shown to reduce the aggressiveness of NSCLC. In particular, the phosphatidylinositol 3-kinase (PI3K)/Akt pathway regulates the expression of NOX4 via nuclear factor-κB (NF-κB), which leads to positive feedback in cell proliferation and invasion in NSCLC [11]. Additionally, Amara et al*. *[12] showed that NOX4 mRNA and protein expression increased in patients with idiopathic pulmonary fibrosis (IPF), and that TGF-β1 increased the expression of NOX4, α-SMA, and procollagen 1 (α1). However, the NOX4 and PI3K pathways have rarely been studied in an ICU model. Erythropoietin-producing hepatocellular receptor A2 (EphA2) is one of the tyrosine kinases that is overexpressed on cell membranes and is expressed at a high level in cancer cells, including NSCLC cells [13]. Menges et al*. *[14] and Park et al*. *[15] showed that EphA2 is linked with the PI3K-Akt pathway in cell cycle arrest.

Based on our understanding of this relationship, it is important to examine the correlation between signaling pathways, including those involving PI3K/Akt, EphA2, and NOX4, in pulmonary injury by ventilators. In this study, we investigated the expression of NOX4 as well as the potential therapeutic effects of a NOX4 inhibitor in a ventilator-induced lung injury (VILI) mouse model and pneumonia patients who used a ventilator.

## Materials and methods

### Experimental animals and groups

All animal experiments were conducted in accordance with recommendations in the Guide for the Care and Use of Laboratory Animals of the National Institutes of Health. All animals were supplied with food and water and were subjected to the same day and night light cycles. The wild-type male C57BL/6J mice (20–28 g; Orient Bio, Sungnam, Korea) and NOX4 knockout (KO) male mice (7–9 weeks 20–28 g, provided by Prof. Ji Hwan Ryu, Severance Biomedical Science Institute, Yonsei University College of Medicine) were divided into the following five groups:

1. Control group: wild-type mice + no ventilation.
2. High tidal ventilation (HTV) group: wild-type mice + HTV ± DMSO 50 μL.
3. Normal ventilation control (NVC) NOX4 KO: NOX4 KO mice + no ventilation.
4. NOX4 KO with HTV group: NOX4 KO mice + HTV.
5. NOX4 inhibitor during HTV group: wild-type mice + HTV + anti-GKT 137831 inhibitor (Additional file 1: Fig S1).

Mice groups using ventilator were anesthetized by intrapenitoneal injection of ketamine (100 mg/kg) and xylazine (10 mg/kg), and additional ketamine was used at about 20–30 min intervals to maintain sedation. Harvard small rodent ventilator (model 683; Harvard apparatus, Holliston, MA, USA)) was used for mechanical ventilation, and mice were ventilated with a high tidal volume of 24 ml/kg and a frequency of 100 breaths/min. For the NOX4 inhibitor during HTV group, 50 μL of DMSO with 50 μg of anti-GKT 137831 inhibitor (BioVision, cat # 9444-5) was injected intraperitoneally into mice at 120 min of ventilation. The mice were euthanized 5 h after the administration of HTV (by a lethal dose of ketamine and xylazine mixture), and their lung tissues were obtained for further analysis.

### Bronchoalveolar lavage

For the BAL fluid (BALF) analysis, BAL was performed with a tracheal cannula and 1 cc of sterile saline. The BALF was centrifuged (4 °C, 3000 rpm, 10 min), and the supernatant was stored at 80 °C for further analysis. The cell pellet was reconstituted in 100 μL phosphate-buffered saline (PBS) and used for cell counts and cytospins. Total cell numbers in each sample were determined using a hemocytometer (Marienfield) according to the manufacturer’s protocol. A 90 μL aliquot of each sample was transferred to chamber slides, which were then inserted into a cytocentrifuge (Shandon Cytospin 4 cytocentrifuge, Thermo Scientific, Waltham, MA, USA) facing outward. The slides were centrifuged at 800 rpm for 5 min, removed from the cytocentrifuge, and dried prior to staining with Diff-Quik Stain Set (Dade Behring, Newark, DE, USA) to assess inflammation. Protein concentrations in the BAL supernatant were determined using a BCA assay (Thermo Fischer Scientific).

### Histopathologic analysis

For the histopathology analysis, left lungs were inflated with low-melting point agarose (4%) in PBS through a tracheotomy incision at an H_2_O pressure of 25 cm until the pleural margins became sharp. The inflation-fixed left lungs of experimental mice were fixed in paraffin and cut to a thickness of 5 μm. After the slides were prepared, they were stained with hematoxylin and eosin, and lung injury scores were determined under light microscopy. These scores were calculated as described by Matute-Bello et al. [16] by using neutrophils in alveolar spaces, neutrophils in interstitial spaces, hyaline membranes, proteinaceous debris filling airspaces, and alveolar septal thickening as a scoring parameters. Lung sections were processed for immunohistochemistry using an anti-NOX4 (UOTRIB492, Abcam) antibody.

### Western blotting and enzyme-linked immunosorbent assay (ELISA)

Lung tissues were harvested and lysed in homogenization buffer (PRO-PREP Extraction solution, iNtRON Biotechnology). The samples were centrifuged at 13,000×*g* for 30 min at 4 °C. The concentrations of proteins in the supernatants were determined by BCA assay (Thermo Fischer Scientific). Equal amounts of protein were separated by sodium dodecyl sulfate–polyacrylamide gel electrophoresis and transferred to a nitrocellulose membrane. Membranes were blocked with 5% skim milk in TBS-T (TBS [170-6435, Bio-Rad Laboratories] and 1% Tween-20 [170-6531, Bio-Rad Laboratories]) for 1 h at room temperature. Then, the membranes were incubated overnight with primary antibody diluted in 5% skim milk and TBS-T at 4 °C. After washing with TBS-T, the blots were incubated with horseradish peroxidase-conjugated secondary antibodies and 5% skim milk in TBS-T for 1 h at room temperature; they were then developed using a Super-Signal West Pico chemiluminescence detection kit (Pierce). The antibodies used in the present study included NOX4 (ab155071, Abcam), EphA2 (PA5-14574, Thermo Fisher Scientific), rabbit PI3 kinase 110γ (Cell Signaling Technologies), and rabbit α-tubulin (PA5-16891, Cell Signaling Technologies). Proteins resolved on western blots were quantified using ImageJ (Image Processing and Analysis in Java, NIH, USA) software.

Interleukin-1β (IL-1β), interleukin-6 (IL-6), and interleukin-8 (IL-8) levels in lung lysates were measured using ELISA kits (Millipore) according to the manufacturer’s directions.

### Real-time polymerase chain reaction (PCR)

Total RNA was isolated from homogenized lungs using a GenElute Mammalian Total RNA Miniprep Kit with DNase treatment. A High Capacity cDNA Reverse Transcription Kit was used to reverse transcribe RNA into cDNA. A real-time PCR analysis of ~ 25 ng cDNA was performed using TaqMan Universal PCR Master Mix for NOX4 (Mm 00479246_m1), GAPDH (Mm 99999915_g1), and pre-designed TaqMan Gene Expression Assays. The reaction was performed on a 7300 Real-Time PCR System (Applied Biosystems, Vienna). Data were analyzed using cyclophilin (Mm 00835365_g1) as a reference gene. No extraneous amplification was confirmed by inclusion of a no-template control.

### Bronchoalveolar lavage fluid from patients with pneumonia

BAL fluid was obtained from 38 patients through bronchoscopy, to examine NOX4 and EphA2 levels in patients with pneumonia. To acquire BALF from patients, a bronchoscope was inserted and wedged through the mouth or nasal route and about 10 cc of BALF was acquired from the patient using 30 ml sterile 0.9% saline. BALF obtained from the opposite site of lung cancer was used as the control group (n = 10), and the NOX4 levels of these patients were compared with the NOX4 levels of BALFs obtained from patients with pneumonia (n = 28). The pneumonia patient group was divided into a group that did not receive ventilator care (n = 10) and a group who received ventilator care (n = 18) in the ICU due to high severity of pneumonia. NOX4 quantification was performed with an ELISA kit (Nori® Human NOX4 ELISA Kit) according to the manufacturer’s instructions. For EphA2 quantification, an ELISA kit (R&D Systems, catalog no. RND-LXSAHM-01, Minneapolis, MN, USA) was used.

### Mechanical stretch-induced lung injury in macrophage

We performed an additional experiment to investigate the roles of EphA2 and NOX4 in alveolar macrophages. The J774A.1 cell line obtained from the ATCC (TIB-67™) was used in a mechanical stretch-induced lung injury model. J774A.1 cells were cultured at a density of 1 × 10^6^ cells/cm^2^ in DMEM + 2 mM glutamine + 10% fetal bovine serum. We used an in vitro mechanical ventilation model using cyclic stretching of alveolar macrophages to mimic high and low tidal volume ventilation strategies. Cells were biaxially stretched in a triangular wave manner for 2 or 4 h at 37 °C using the FX-6000 Tension System (Flexcell, Burlington, NC, USA). Thirty cycles/min, with a stretch/relaxation relation of 1:1, and 20% changes in the basement membrane surface area were applied for mechanical stretching.

We measured the levels of IL-6 (Koma Biotech, catalog no. K0331230HS, Seoul, South Korea) and IL-8 (Invitrogen, catalog no. EMCXCL1, Waltham, MA, USA) with and without EphA2 (10 µM) or NOX4 (10 µM) inhibition. We subjected the macrophages to the following treatments:

1. No treatment (control).
2. Mechanical stretch for 2 or 4 h.
3. EphA2 inhibition (1 h before cell stretch) + Mechanical stretch.
4. NOX4 inhibition (1 h before cell stretch) + Mechanical stretch.

### Statistical analysis

A statistical analysis was performed in Prism version 5.0 (GraphPad Software, Durham, NC, USA), and the data are expressed as mean ± standard deviation. Comparisons between groups were made using a two-way analysis of variance (ANOVA) and corrected using the Bonferroni correction method. p < 0.05 was considered significant.

## Results

### NOX4 KO or NOX4 inhibitor attenuate VILI

**Fig. 1:**
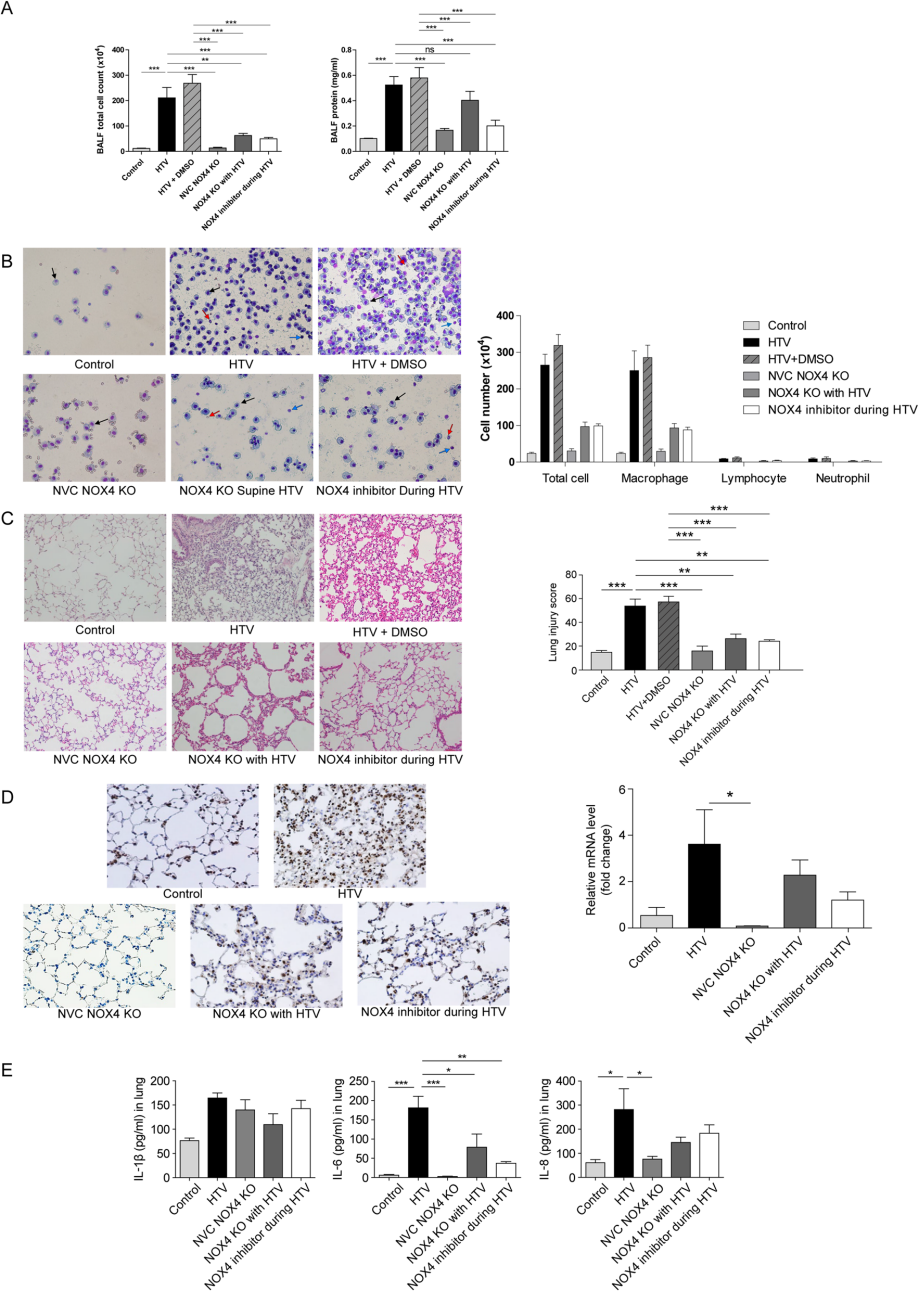
NOX4 KO and NOX4 inhibitor attenuates VILI in mice. **A** Total cell counts and protein concentrations. **B** Cell differentiation in BALF with a representative visual field. Cells were mainly macrophages (black arrow) with a few lymphocytes (blue arrow) and neutrophils (red arrow). **C** Histopathologic features of hematoxylin and eosin-stained mouse lung tissues and lung injury score showing the extent of vascular engorgement, hyaline membrane formation, alveolar wall edema, and inflammatory cell infiltration (200 × magnification). **D** Relative levels of NOX4 mRNA determined by real-time PCR (n = 3, each group) and NOX4 immunohistochemistry (400 × magnification). **E** Inflammatory cytokine levels; IL-1β, IL-6, and IL-8 levels in lung tissue lysates. N = 8 in each group. *p < 0.05, **p < 0.01, and ***p < 0.001, analyzed by two-way ANOVA with post-hoc testing and Bonferroni correction. *BALF* bronchoalveolar lavage fluid, *NVC* normal ventilation control, *KO* knockout, *HTV* high tidal ventilation

As shown in Fig. 1, cell counts and protein concentrations for brochoalveolar lavage fluid (BALF) bronchial cells were significantly higher in the HTV ± DMSO group than in the NOX4 inhibitor during HTV group (p < 0.001, Fig. 1A). Cell counts were significantly lower (p < 0.01) and protein concentrations tended to be lower in the NOX4 knockout (KO) with HTV group than in the HTV group.

Figure 1B shows the differential cell counts from cytospins. Macrophages were predominant, and there were few lymphocytes in the BALF from the HTV ± DMSO group. Additionally, in the histopathologic analysis (Fig. 1C), the extents of leukocyte infiltration, capillary leakage, hyaline membrane formation, and alveolar wall edema were found to be the greatest in the HTV ± DMSO group. NOX4 mRNA levels were higher in the HTV group, followed by the NOX4 KO with HTV, NOX4 inhibitor, control, and NOX4 KO groups (Fig. 1D). NOX4 mRNA levels tended to be lower in the NOX4 group than in the HTV group. Increased NOX4 immunostaining after HTV was reduced by NOX4 inhibition (Fig. 1D).

Cytokine levels were measured by ELISA in lung tissue lysate. The cytokine concentrations of IL-6 and IL-8 were significantly higher in the HTV group than in the control group (Fig. 1E), and the expression of IL-6 was significantly decreased in both of the NOX4 KO (p < 0.05) and NOX4 inhibitor groups (p < 0.01).

### VILI decreased with downregulation of NOX4, EphA2, and PI3K 110λ signaling

**Fig. 2:**
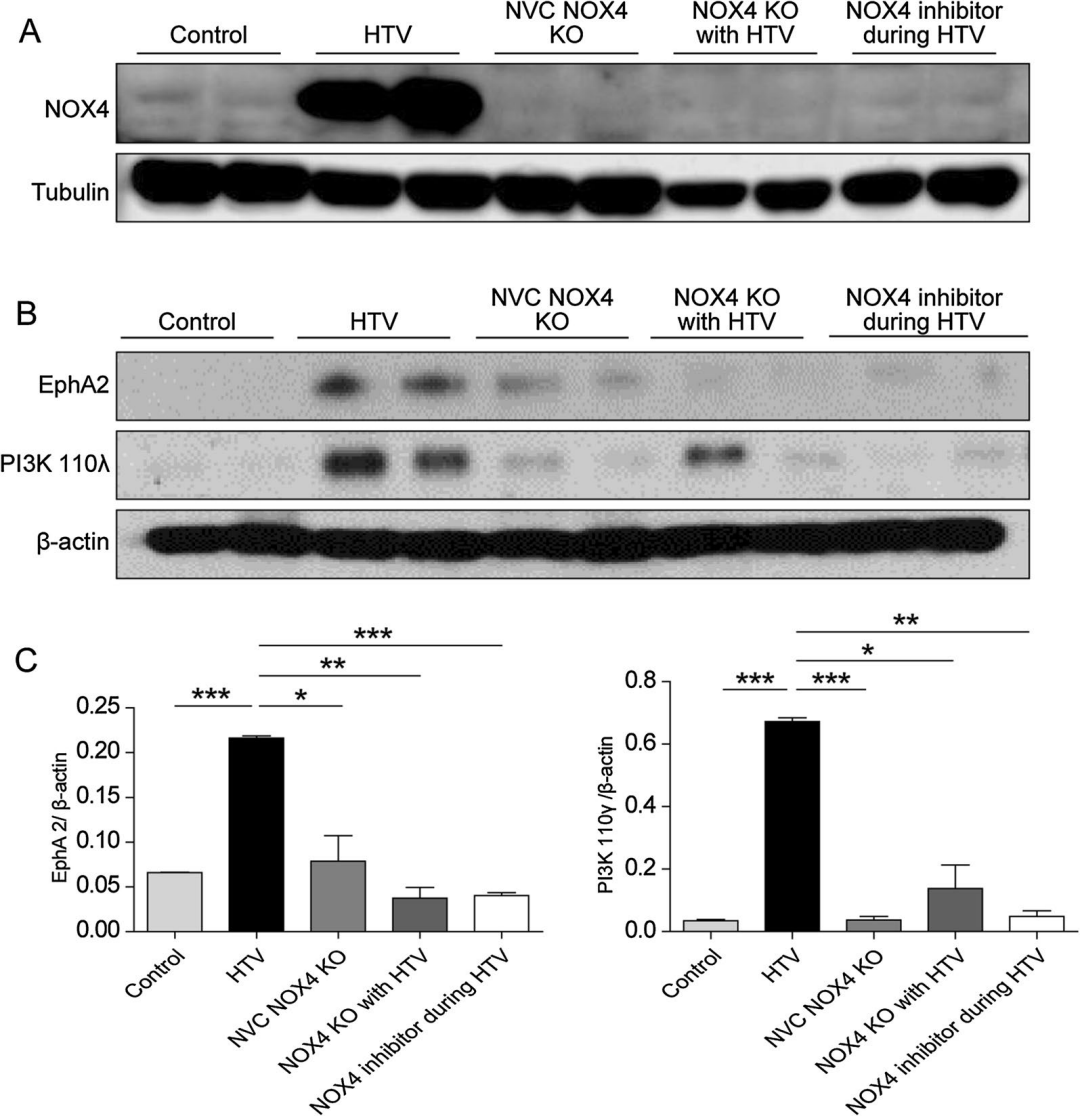
VILI is decreased in the NOX4 KO mouse and NOX4 inhibitor groups. Western blot of NOX4 (**A**), EphA2, and PI3K 110λ (**B**). **C** Densitometry of EphA2, and PI3K 110λ. *p < 0.05, **p < 0.01, and ***p < 0.001, analyzed by two-way ANOVA with post-hoc testing and Bonferroni correction. *KO* knockout, *HTV* high tidal ventilation

To further evaluate the effects of NOX4 inhibition on VILI, we examined NOX4, EphA2, and PI3K 110λ signaling by western blot analysis (Fig. 2). NOX4, EphA2, and PI3K 110λ expression levels were significantly higher in the HTV group compared to those in the control group. Furthermore, in both the NOX4 KO group and NOX4 inhibitor group, the expression of these signaling molecules was significantly lower than that in the HTV group (Fig. 2A). Treatment with the NOX4 inhibitor during HTV significantly attenuated VILI. These results indicated that the NOX4, EphA2, and PI3K 110λ signaling pathways are involved in VILI, and that a NOX4 inhibitor could be a therapeutic potential agent in the treatment of ALI/ARDS in VILI.

### VILI upregulates EphA2 via NOX4 activation

**Fig. 3:**
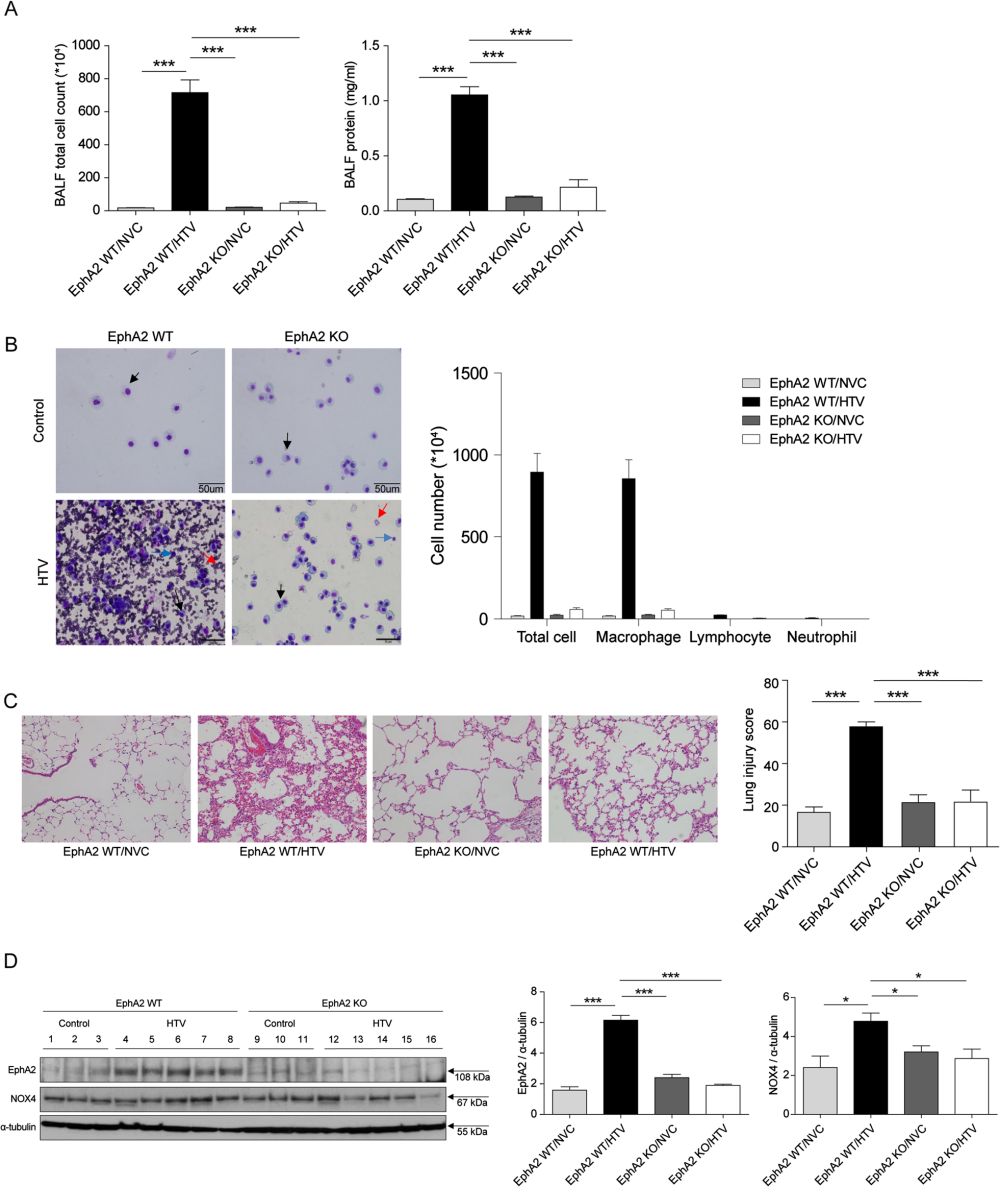
NOX4 is associated with EphA2 expression in VILI model. **A** Total cell counts and protein concentrations. **B** Identification of cells in BALF with representative visual fields. Cells were mainly macrophages (black arrow) with a few lymphocytes (blue arrow), and neutrophils (red arrow). **C** Histopathologic features of hematoxylin and eosin-stained mouse lung tissues and lung injury score, showing vascular engorgement, hyaline membrane formation, alveolar wall edema, and inflammatory cell infiltration (200 × magnification). **D** Western blot of EphA2 and NOX4, and densitometry of EphA2 and NOX4 in response to HTV and EphA2 KO. N = 3 in NVC group and n = 5 in HTV group. *p < 0.05, **p < 0.01, and ***p < 0.001, analyzed by two-way ANOVA with post-hoc testing and Bonferroni correction. *BALF* bronchoalveolar lavage fluid, *WT* wild-type, *KO* knockout, *NVC* normal ventilation control, *HTV* high tidal ventilation

Given the protective effect of the NOX4 inhibitor on VILI, we postulated that NOX4 mediates VILI through an EphA2-dependent pathway. Therefore, we investigated the expression of NOX4 and EphA2 using EphA2 KO mice. First, we examined the effects of EphA2 on VILI (Fig. 3). Cell counts and protein concentrations in BALF were significantly higher in the HTV groups and lower in the EphA2 KO groups (both p < 0.001; Fig. 3A). BALF cells mainly included macrophages; a few lymphocytes were present in the wild-type (WT)/HTV group but not the EphA2 KO/HTV group (Fig. 3B). Similarly, histopathologic analysis showed that the extent of lung injury was the most severe in the HTV group and less severe in the EphA2 KO/HTV group (p < 0.001; Fig. 3C).

Figure 3D shows the results of the western blot and densitometry analyses of EphA2 and NOX4 protein levels. EphA2 expression was significantly higher in the HTV group and lower in EphA2 KO groups, even with ventilation (p < 0.001; Fig. 3D). NOX4 expression was significantly higher in the HTV group, but it decreased in both the unventilated and ventilated EphA2 KO mice groups (both p < 0.05; Fig. 3D). These results showed that NOX4 and EphA2 signaling are involved in VILI, and that EphA2 is activated through a NOX4-dependent pathway.

### NOX4/EphA2 levels are increased in patients with pneumonia

**Fig. 4:**
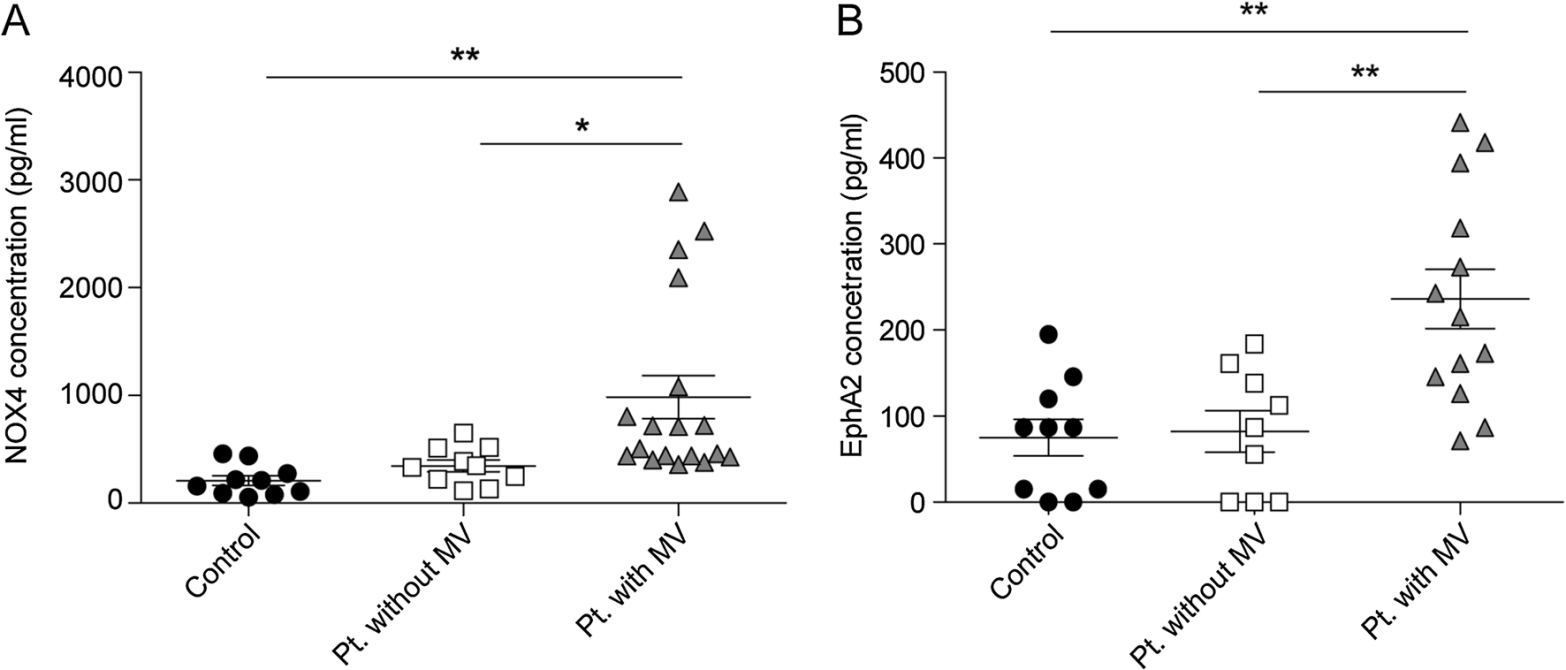
NOX4/EphA2 level was significantly elevated in pneumonia patients, especially in pneumonia patients using ventilator. **A** NOX4 concentration. **B** EphA2 concentration. *p < 0.05, and **p < 0.01, analyzed by two-way ANOVA with post-hoc testing and Bonferroni correction. *Pt* patients, *MV* mechanical ventilator

The NOX4 and EphA2 levels in BALF were investigated in patients with/without pneumonia. The baseline characteristics of the patients are shown in Table 1. Median age, gender, and BMI were not significantly different between the three groups, but the in-hospital mortality rate was significantly higher in the patient group receiving ventilator care than in the other groups (p = 0.005). The NOX4 and EphA2 levels were significantly higher in patients with pneumonia compared with those in the control group. Among the pneumonia patients, NOX4 levels were highest in the group that received mechanical ventilator care (Fig. 4, p < 0.01 compared with that in the control group, and p < 0.05 compared with that in pneumonia patients without ventilation). EphA2 levels were also highest in the ventilated patient group (p < 0.01, compared with that in the control group).

**Table 1:** Clinical characteristics of study patients

	Control (n = 10)	Pneumonia without ventilator care (n = 10)	Pneumonia with ventilator care (n = 18)	*p*-value
Age, years, median (IQR)	63.0 (49.3, 72.5)	47.5 (37.8, 51.5)	63.5 (44.8, 74.3)	0.070
Gender, male, N (%)	6 (60%)	6 (60%)	11 (61.1%)	0.998
BMI (kg/m^2^), median (IQR)	24.1 (19.9, 26.3)	20.8 (16.9, 22.1)	22.9 (20.2, 24.9)	0.112
Length of stay, d, median (IQR)	2.0 (0.0, 3.3)	37.5 (20.8, 56.0)	34.5 (28.5, 66.3)	< 0.001
28-day mortality, N (%)	0	0	4 (22.2)	0.083
In-hospital mortality, N (%)	0	3 (30%)	11 (61.1%)	0.005

*IQR* interquartile range, *BMI* body mass index

### Effect of NOX4/EphA2 inhibition in alveolar macrophage

We assessed the levels of IL-6 and IL-8 by EphA2 or NOX4 inhibition in alveolar macrophages. Cell stretch was significantly associated with increased levels of IL-6 and IL-8, and the expression of IL-6 and IL-8 was significantly decreased in the EphA2 and NOX4 antibody pretreated cells (Additional file 1: Fig S2).

## Discussion

For patients with ARDS, a ventilator is essential for supplying oxygen, but it can also cause lung injury. In this study, we developed a VILI model. Then, we examined the role of NOX4 and the effects of NOX4 inhibition and as the blockade of EphA2 using KO mice in this VILI model. We also examined NOX4 levels in patients with respiratory diseases according to their use of a ventilator.

NOX4 acts as an oxygen sensor for catalysis of molecular oxygen in various reactive oxygen species (ROS), and the resulting ROS undergo many biological reactions, including those involved in signal transduction, cell differentiation, and tumor cell growth [17]. Eph-ephrin signaling, as part of receptor tyrosine kinase (RTK) pathways, have been implicated in many cellular processes, including vasculogenesis, angiogenesis, cell migration, axon guidance, embryonic development, fluid homeostasis, and injury repair [18, 19].

Palumbo et al. [20] reported the role of NOX4 in the lungs and showed that ALI is more severe in aged mice than in young mice because of increased vascular permeability, albumin influx, and BAL neutrophils and proteins. In addition, they showed that ROS levels are elevated in aged or injured mice, suggesting that lung injury is associated with NOX4, a ROS-generating enzyme. They detected the senescence of endothelial cells based on β-galactosidase activity and an increase in p16 level and studied the regulation of the endothelial cell barrier in human lungs. Their results showed that membrane permeability caused by LPS is increased in senescent endothelial cells compared to that in young endothelial cells, and the expression of NOX4 is rapidly induced by LPS challenge via a proteasome/ubiquitin system. They found that pharmacological inhibition of NOX4 reduced the change in membrane permeability due to LPS. Our study showed that NOX4 was involved in VILI, similar to its involvement in lung injury by LPS. Our results also demonstrated lung injury prevention by NOX4 inhibition.

Hong et al. [21] reported downregulation of PI3K 110γ, phospho-Akt, phospho-NF-κB p65, phospho-Src, and phospho-S6K via EphA2 antibody in an LPS-induced lung injury model. The above two studies showed that NOX4 and EphA2 are involved in lung injury due to LPS and that NOX4 inhibition and EphA2 receptor inhibition are effective in limiting such lung injury. Therefore, with VILI, it can be inferred that the NOX4 and EphA2 pathways function similarly to that in LPS-induced lung injury, and that there is a therapeutic effect from NOX4 or EphA2 inhibition.

A study of the EphA2/ephrinA1 signaling pathways in a VILI model was performed by Park et al. [15] Placing mice in a prone position reduced lung injury, and EphA2 antagonism downregulated the expression of PI3Kγ, Akt, and NF-κB, which resulted in lung protective effects. EphA2/ephrinA1 is shown to be a VILI therapeutic target in their study. Similarly, Leem et al. [22] reported that EphA2/ephrinA1 is elevated by bleomycin in a bleomycin-induced lung injury model, that elevation of IL-6 and TNF-a in PI3K-Akt leads to lung injury, and that elevation of EphA2-ephrin A1 could be blocked by all-trans retinoic acid. As indicated by the studies described above, EphA2 levels are closely related to the extent of lung injury in patients undergoing ventilator care in ICU, and, in our study, we found that EphA2 levels were higher in the severe lung injury group. Che et al. [23] showed that bleomycin-induced lung injury is caused by TGF-β/Smad3 signaling and oxidative stress, which can be regulated by a Chinese medicine called Shenks. This medicine increases the expression of the antioxidant-related genes Gclc and Ec-sod, both in vivo and in vitro, by increasing the transcription of oxidative-related genes, including Rac1 and Nox4. Zhang et al. [24] also showed the overexpression of NOX4 in a bleomycin-induced lung injury model and reported that schizandrin B and glycyrrhizic acid are effective inhibitors of the TGF-β1/Smad3 signaling pathway in this model.

Lee et al. [25] measured plasma EphA2 levels in a prospective study of patients who were admitted to an ICU because of sepsis and had their disease severity determined based on acute physiology and chronic health evaluation (APACHE) II and sequential organ failure assessment (SOFA) scores to examine the correlation between SOFA score and EphA2 level. They confirmed a positive correlation between serum levels of the EphA2 receptor and severity of sepsis in ICU patients. In addition, when the area under the curve of EphA2 receptor levels is measured, it is 0.690 higher than that of the APACHE II scores. Additionally, they showed that EphA2 receptor levels are associated with sepsis severity and 28-day mortality. These results suggest that EphA2 levels are associated with severity of VILI in ICU patients.

NOX4 is also expressed in the pulmonary endothelium, which acts as a barrier that prevents plasma exudate from entering the interstitium and alveolar space, and plays an important role in regulating lung inflammation, apoptosis, and permeability along with NOX2 in pneumonia caused by *Pseudomonas aeruginosa* [26]*.*

BAL is a commonly used procedure for evaluating various lung diseases in mice and humans. It can be used for assessing environmental toxin exposure, microorganisms, and pathophysiological conditions. Additionally, many previous studies have shown that BAL can be used to describe the tumor microenvironment, indicating that BALF represents the environment of respiratory diseases [27, 28]. In our study, NOX4/EphsA2 levels in BALF were also high in patients with pneumonia. In particular, NOX4 levels were significantly higher in the group of pneumonia patients using ventilators in the ICU, matching the data derived from the mouse experiments.

In summary, we found that a signaling pathway with NOX4, EphA2, and PI3K is associated with VILI and that there are several potential mechanisms by which NOX4 inhibition may affect VILI. In addition, we showed the potential for a NOX4 inhibitor to decrease VILI through EphA2 and PI3k 110λ signaling (Additional file 1: Fig S3).

## Conclusion

In this study, we showed that NOX4 and Eph-ephrin signaling is involved in VILI and that a NOX4 inhibitor can play a therapeutic role in VILI. Further studies with human samples are needed to investigate the role of NOX4 signaling in VILI.

## Supplementary Information

**Additional file 1:** **Fig. S1**. Time table for NOX4 inhibitor treatment in the high tidal volume group. **Fig. S2**. Effect of NOX4/EphA2 inhibition in alveolar macrophage under mechanical stretch-induced lung injury. **Fig. S3**. Potential mechanisms by which NOX4 inhibition attenuates VILI.

## Data Availability

The data that support the findings of this study are available from the corresponding author upon reasonable request.
