# Supplementary material for: NADPH oxidase 4 signaling in a ventilator-induced lung injury mouse model
Source: Respir Res. 2022 Mar 27;23:73. doi: 10.1186/s12931-022-01992-0 (PMC8962540; doi:10.1186/s12931-022-01992-0)

- **Supplementary data**

**NADPH oxidase 4 signaling in a ventilator-induced lung injury mouse model**

Sang Hoon Lee, Mi Hwa Shin, Ah Young Leem, Su Hwan Lee, Kyung Soo Chung, Young Sam Kim, Moo Suk Park

Division of Pulmonology, Department of Internal Medicine, Institute of Chest Diseases, Severance Hospital, Yonsei University College of Medicine, Seoul, Republic of Korea

**Fig S1.** **Time table for NOX4 inhibitor treatment in the high tidal volume group**

In the VILI model, mice were tracheostomized, and the ventilator was maintained for 5 h in the supine position with 24 mL/kg volume, 0 cmH_2_O PEEP, 100/min respiration rate, and 0.21 inspired oxygen fraction.

**
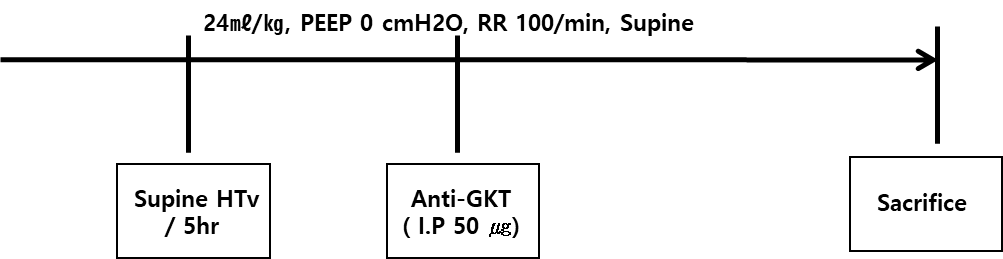
**

**Fig S2. Effect of NOX4/EphA2 inhibition in alveolar macrophage under mechanical stretch-induced lung injury**

(A) IL-6 and (B) IL-8 levels *p < 0.05, **p < 0.01, and ***p < 0.001, analyzed by two-way ANOVA with post-hoc testing and Bonferroni correction.

**Fig S3. Potential mechanisms by which NOX4 inhibition attenuates VILI.** Our study suggests that NOX4 inhibition decreases VILI through EphA2, PI3K-r, and IL-6 signaling.

VILI, ventilator-induced lung injury


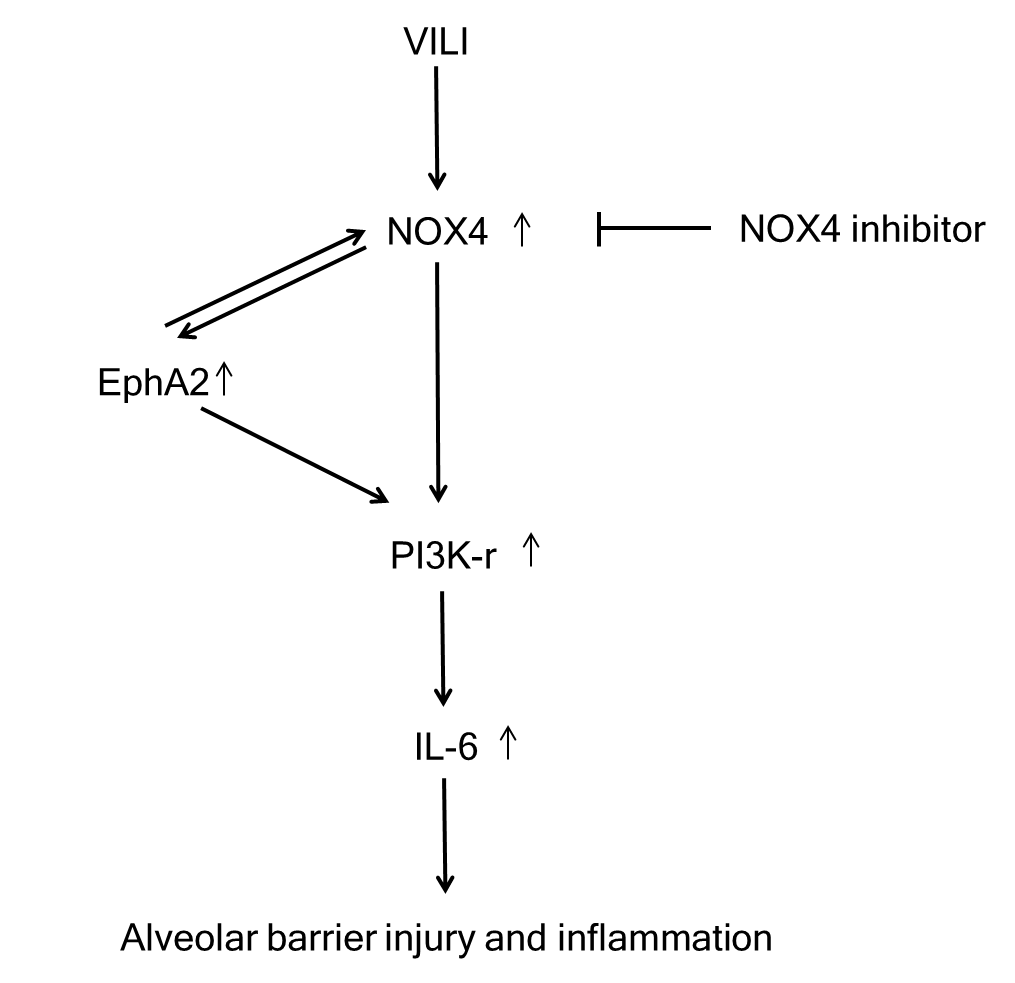

Supplement: Supplementary file 1 — Additional file 1: Fig. S1. Time table for NOX4 inhibitor treatment in the high tidal volume group. Fig. S2. Effect of NOX4/EphA2 inhibition in alveolar macrophage under mechanical stretch-induced lung injury. Fig. S3. Potential mechanisms by which NOX4 inhibition attenuates VILI. [file 12931_2022_1992_MOESM1_ESM.docx]
